# Highly complicated lead extraction procedure in patient with previous early orthotopic heart transplantation: A first case report

**DOI:** 10.1002/ccr3.1823

**Published:** 2018-10-19

**Authors:** Vincenzo Schillaci, Giuseppe Mascia, Gergana Shopova, Paola Chiariello, Carmine Ciardiello, Francesco Solimene

**Affiliations:** ^1^ Electrophysiology Unit Clinica Montevergine Mercogliano Italy; ^2^ Cardiology Department Ospedale Cava de Tirreni Italy; ^3^ Clinical Affairs HT MED Pozzuoli ITALY

**Keywords:** device implantation, heart failure, lead extraction, orthotopic heart transplantation

## Abstract

We report a first case of a highly complicated lead extraction in a young man who previously underwent orthotopic heart transplantation (OHT).Lead extraction in transplanted patients may be a feasible and safe procedure in order to maintain a low infective risk and to preserve alternative vascular access sites.

## 1 CASE REPORT

End‐stage heart failure patients with a previous cardiac transplantation could potentially require an implantable cardioverter‐defibrillator (ICD) or cardiac resynchronisation therapy (CRT) device implantation. We report a first case of a highly complicated lead extraction and subsequent device reimplantation in a young man who previously underwent orthotopic heart transplantation (OHT). Actually, it is still unknown the best lead extraction approach in this difficult patient population, since to date no cases have been described in medical literature. Our case report establishes an important strategy model in this category of "high‐risk" patients.

A 33 years old male came to our attention in August 2017. The patient underwent OHT in 1997 (13 years of age) due to arrhythmogenic biventricular dysplasia/cardiomyopathy and dilated‐hypokinetic evolution. He was affected by hypertension, dyslipidemia, and slight renal impairment. He was in treatment with antihypertensive drugs and immunosuppressive therapy (cyclosporine, azathioprine, and prednisone) for triple rejection (2004, 2009, and 2012, respectively). In 2004, during his first rejection episode, a coronary angiography was performed, documenting normal coronary angiograms. In 2012 (at 28 years of age), the patient, at first, developed fatigue and dyspnea. An echocardiogram documented left ventricular dysfunction with severe reduction in global contractility, ejection fraction (EF) 35%, and mechanical dyssynchrony. The electrocardiogram (ECG) showed sinus rhythm with narrow QRS. In the same year (2012), a cardiac resynchronization therapy defibrillator (CRT‐D) Boston Scientific Cognis 100‐D was surprisingly implanted. Right ventricular (RV) apex lead was Boston Scientific Reliance 4‐front 0693 single coil, active fixation; right atrial (RA) lead was Medtronic CapSure Z Novus 5554 bipolar, active fixation; left ventricular (LV) lead was Medtronic Attain Ability 4196 bipolar. In August 2017 (at 33 years old), the patient came to our attention for the first time because of RV lead dysfunction. The ventricular lead impedance was >3000 ohms, and no capture was noted at maximal output. At admission in our department, the patient was asymptomatic. The ECG showed sinus bradycardia, while echocardiogram revealed a markedly dilated left ventricle, severe reduction in global contractility (EF: 32%), restrictive filling pattern (grade 3 diastolic dysfunction), and moderate mitral regurgitation. Chest X‐ray was normal. Blood glucose level was 220 mg/dL, creatinine 1.6 mg/dL, white blood cell (WBC) 11.04 × 10^3^/µL. Interrogation of device confirmed an RV lead impedance value>3000 ohms and concomitant loss of capture, with normal RA and LV impedance values. Therefore, we decided at first to perform RV lead revision.

## 2 PROCEDURE

**Figure 1:**
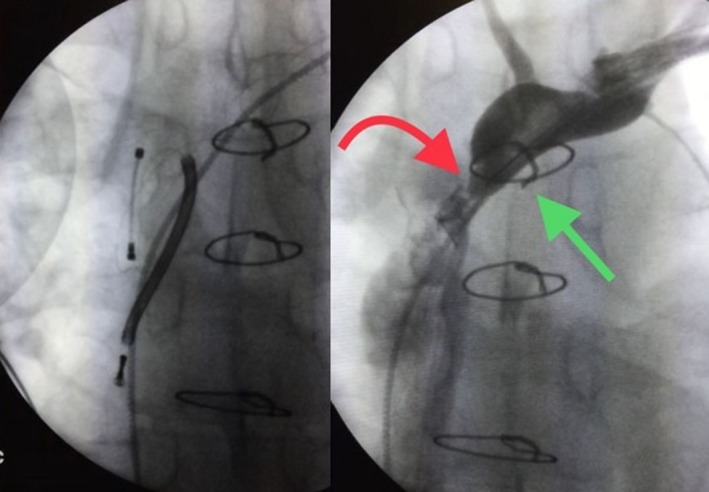
Leads fracture at obstruction level. Red arrow shows obstruction, green arrow shows liberator stylet

**Figure 2:**
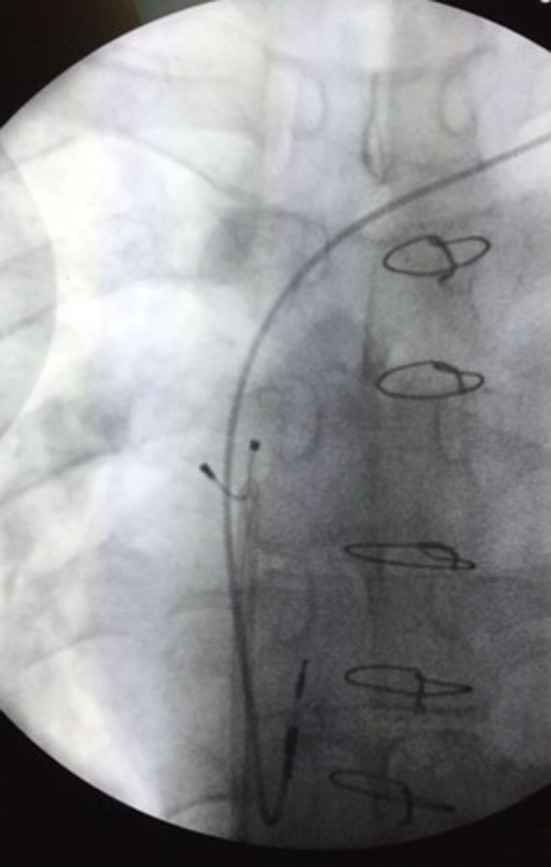
Femoral extraction of the broken left ventricular lead

After obtaining written informed consent, an invasive hemodynamic monitoring through an arterial line and a temporary transvenous pacing through femoral vein were placed. The procedure was performed under general anesthesia (propofol 1% 3 mg/kg/h, fentanyl 300 μg) and pre‐emptive analgesia (tramadol 100 mg, metoclopramide 10 mg, ketorolac 30 mg, paracetamol 1 g, ranitidine 50 mg, cefazolin 2 g)with an active cardiothoracic surgery backup. Defibrillator pocket region was explored, and CRT‐D generator was explanted. An inspection surprisingly revealed that both RV and LV leads were fractured in multiple points. The patency of leads lumen was checked by a standard stylet after the lead's dissection from fibrous tissue, until the subclavian vein puncture site. Then, we performed selective angiography of the left subclavian vein documenting obstruction of the brachiocephalic vein (Figure 1). A stepwise extraction approach was used. RV lead active fixation was unscrewed from the endocardium, and a locking stylet (Liberator Cook Medical, Bloomington, IN, USA) was exchanged with the standard one. The locking stylet was hardly advanced till obstruction site, and simple traction was attempted. The simple traction proved unsuccessful, and a new attempt was performed using a laser dilator sheath advanced over the lead/locking stylet complex (Spectranetics 14 Fr, Colorado Springs, CO). However, it was not possible to advance the laser dilator sheath over the obstruction due to the presence of important calcification at superior vena cava/junction of the brachiocephalic veins. Moreover, during the lead traction, a distal coil migration to the cavoatrial junction (CAJ) occurred, and RV lead fractured proximally to the obstruction. About LV lead removal, the locking stylet (Liberator Cook Medical) was not advanced to the distal portion due to multiple lead's fractures, and a bulldog lead extender system (Cook Medical) was used for the fixation of lead. A laser dilator sheath (Spectranetics, 14 F) was used to perform the LV lead extraction, but again a distal lead migration to the CAJ occurred, and LV lead fractured proximally. Finally, the patient had a good RA lead performance, and accordingly a good site in the right atrial appendage (RAA) was found. At this point, we decided to stop the procedure in order to achieve the optimal strategy. At the end of procedure, the echocardiogram revealed no pericardial effusion. Chest X‐ray confirmed the presence of two fragments, and blood examinations documented WBC 13.91 × 10^3^/µL and hemoglobin 11.8 g/dL. Two days later, whether also the WBC and the hemoglobin were within the reference range, a femoral approach lead extraction was performed advancing a 16 French long sheath through the right iliac vein (Needle's Eye Snare®, Cook Medical) with complete procedural success (Figure 2). A complete procedural success was achieved according to the Heart Rhythm Society (HRS) consensus on lead management.^1^ One day later, based on clinical evaluation and normal laboratory test results, we performed again a selective venography of the left subclavian vein, documenting patency till superior vena cava. Consequently, the implantation of two‐chamber ICD (Medtronic Evera DR MRI safe scan) was performed. RA lead was the pre‐existent Medtronic CapSure Z Novus 5554 bipolar; RV apex lead was Medtronic Sprint‐Quattro 6935 M single‐coil. RV lead was implanted through the patency created by the leads removal. At hospital discharge, the patient remained asymptomatic with normal echocardiogram, chest X‐ray, and blood examinations values.

## 3 DISCUSSION

To date, no lead extraction cases of patients who previously underwent to heart transplant have been described in the medical literature, while on the other side an increasing number of patients is referred for OHT after a previous ICD/CRT device implantation.^2^, ^3^, ^4^, ^5^ Our case report establishes a strategy model in this category of "high risk" patients. The difficulties involved both the procedural approach due to many postsurgical lead's adherences, and also the high risk of infection especially due to concomitant immunosuppressive therapy. In the decision‐making process between the abandonment of malfunctioning RV/LV leads and the extraction of both, a lead extraction strategy prevailed due to the patient's long life expectancy and the pre‐existing presence of three leads in the vascular system.^6^, ^7^ Therefore, we proceeded to RV and LV leads extraction through a subclavian approach due to: (a) both RV and LV leads malfunction; (b) no subclavian vein access; (c) higher risk of infection in case of implantation of new leads on the contralateral side and tunneling of these to the ipsilateral pocket, due to the presence of abandoned leads. We used a laser sheath removal system in order to preserve the normal functioning of RA lead and because of the difficulty of traction along the lead's course without any support (locking stylet partly introduced). After failed leads extraction, we had two options: (a) a new contralateral implantation abandoning both RV and LV leads and tunneling RA lead; (b) lead extraction via a femoral approach. The femoral lead extraction allowed to safely complete the procedure and to preserve the vascular system for a subsequent device implantation. Finally, a dual‐chamber device was implanted due to the lack of response to resynchronization therapy and the absence of indications showing the ECG a sinus rhythm with narrow QRS. Our clinical case report underlines how lead(s) extraction procedures of complex patients with high life expectancy should be always performed by an experienced team, with all possible instruments, allowing the physician to choose the best strategy to achieve procedural success. Lead extraction in transplanted patients may be a feasible and safe procedure in order to maintain a low infective risk and to preserve alternative vascular access sites.

## 4 TEACHING POINTS

- This is a first lead extraction case of patient who previously underwent to heart transplant, establishing a strategy model in this category of "high risk" patients.
- Lead extraction in transplanted patients may be a feasible and safe procedure
- Lead extraction procedures of complex patients should be always performed with all possible instruments, allowing the physician to choose the best strategy to achieve procedural success.

## CONFLICT OF INTEREST

None declared.

## AUTHOR CONTRIBUTION

VS, GM, GS, PC,CC, FS: All six authors contributed equally to this case. In particular: VS and FS performed the procedure. CC supported the procedure. VS, GM, and FS equally contributed to write the manuscript. GS and PC performed routinely patient’s follow‐up at our institution
